# The first 500 days of life: policies to support maternal nutrition

**DOI:** 10.3402/gha.v7.23623

**Published:** 2014-06-06

**Authors:** John B. Mason, Roger Shrimpton, Lisa S. Saldanha, Usha Ramakrishnan, Cesar G. Victora, Amy Webb Girard, Deborah A. McFarland, Reynaldo Martorell

**Affiliations:** 1 Department of Global Community Health and Behavioral Sciences, School of Public Health and Tropical Medicine, Tulane University, New Orleans, LA, USA; 2 Hubert Department of Global Health, Rollins School of Public Health, Emory University, Atlanta, GA, USA; 3 Department of Social Medicine, Faculty of Medicine, Universidade Federal de Pelotas, Pelotas, Brazil

**Keywords:** maternal nutrition, women's health, intrauterine growth restriction, anemia, nutrition interventions

## Abstract

**Background:**

From conception to 6 months of age, an infant is entirely dependent for its nutrition on the mother: via the placenta and then ideally via exclusive breastfeeding. This period of 15 months – about 500 days – is the most important and vulnerable in a child's life: it must be protected through policies supporting maternal nutrition and health. Those addressing nutritional status are discussed here.

**Objective and design:**

This paper aims to summarize research on policies and programs to protect women's nutrition in order to improve birth outcomes in low- and middle-income countries, based on studies of efficacy from the literature, and on effectiveness, globally and in selected countries involving in-depth data collection in communities in Ethiopia, India and Northern Nigeria. Results of this research have been published in the academic literature (more than 30 papers). The conclusions now need to be advocated to policy-makers.

**Results:**

The priority problems addressed are: intrauterine growth restriction (IUGR), women's anemia, thinness, and stunting. The priority interventions that need to be widely expanded for women before and during pregnancy, are: supplementation with iron–folic acid or multiple micronutrients; expanding coverage of iodine fortification of salt particularly to remote areas and the poorest populations; targeted provision of balanced protein energy supplements when significant resources are available; reducing teenage pregnancies; increasing interpregnancy intervals through family planning programs; and building on conditional cash transfer programs, both to provide resources and as a platform for public education. All these have known efficacy but are of inadequate coverage and resourcing. The next steps are to overcome barriers to wide implementation, without which targets for maternal and child health and nutrition (e.g. by WHO) are unlikely to be met, especially in the poorest countries.

**Conclusions:**

This agenda requires policy decisions both at Ministry and donor levels, and throughout the administrative system. Evidence-based interventions are established as a basis for these decisions, there are clear advocacy messages, and there are no scientific reasons for delay.

The ‘first 1,000 days of life’, a critical period for growth and development, has become a global rallying cry.^1^ This has resulted in greater attention to children under two, including efforts to ensure safe delivery and increase survival, and the promotion of appropriate infant and young child feeding practices. Actions have been directed more to the child than to the mother. For the first 500 of these days, from conception to about 6 months of age, the infant is entirely dependent for its nutrition on the mother: via the placenta and then ideally via exclusive breastfeeding.

Although indisputably central to early life, maternal nutrition has only recently begun to be highlighted in government or agency policies and activities (1, 2). In 2012, the World Health Assembly endorsed the WHO ‘Comprehensive implementation plan on infant and young child nutrition’ which now prioritizes women's nutrition, anemia, intrauterine growth retardation (IUGR) and low birth weight (LBW) (3, 4) (paras 2–4). Moreover, there are now global targets:

1. ‘Global target 2: by 2025, a 50% reduction of anemia in women of reproductive age’;
2. ‘Global target 3: by 2025, a 30% reduction of LBW’; and
3. ‘Global target 5: by 2025, increase the rate of exclusive breastfeeding in the first 6 months up to at least 50%’.

This paper adds its voice to the attention to the first 1,000 days. By pointing to the earliest of these days, the first 500 days, we stress the unique role of maternal nutrition in moving towards WHO's targets.

We recently carried out a landscaping study of programs addressing maternal nutrition and birth outcomes, both globally and with country case studies (Ethiopia, India, and Northeast Nigeria): these included more than 100 focus group discussions, 140 key informant interviews, plus extensive literature searching and informal discussions with officials of government, UN, and non-governmental organizations (NGOs) (5–9). We focused on three major problems: maternal anemia, IUGR, and small body size (thinness and stunting) in adult women – much the same as in the WHO plan.

Nutrition problems affect half or more of women in many low- and middle-income countries (LMICs), with disproportionate risk among the poorest (10). In LMICs overall, anemia prevalence averages 40%, affecting 600 million women, with limited progress seen in the last two decades (11, 12). Anemia prevalence in women of reproductive age exceeds 50% in many parts of India and Nigeria, and is 17% in Ethiopia (6–8). The prevalence of thinness (body mass index (BMI) <18.5) in rural women is up to 28% in Ethiopia, 41% in India, and 14% in Nigeria (13). Current estimates of short stature (<150 cm, which is −2 Standard Deviations of height-for-age) are less widely available; existing national estimates are 39% for India, 13% for Ethiopia and 3% for Nigeria (6, 8, 14).

The effects of maternal malnutrition on the developing child *in utero* are extensive. Risk of IUGR and prematurity are increased in the offspring of women who are underweight at the beginning of pregnancy (15). There is increasing evidence that inadequate fetal growth and development (i.e. IUGR) can have lifelong effects. The fetal origins of disease, described in 1986 by Barker et al. (16) and the subject of much research since then (17, 18), through impaired glucose tolerance and other paths are a risk for metabolic syndrome and premature mortality in mid-life; this suggests an increasing burden for health services in poor but rapidly changing countries, where alterations in lifestyles and diets exacerbate this effect (11). Neurocognitive and related development is at risk from ‘intrauterine and neonatal insults’, with serious long-term implications (19). IUGR precedes and contributes to stunting, now a widely used biomarker of child growth and development (11). Maternal anemia during pregnancy increases the risk of prematurity and LBW, as well as of low iron stores in newborns (20). In turn, low iron stores at birth are a risk factor for postnatal iron deficiency and anemia, which impairs motor and mental development, and learning (21). Unless this malnutrition among women is addressed, malnutrition among children will not be overcome.

Malnutrition adversely affects women's health itself. Low BMI is associated with lethargy, reduced physical activity, immunosuppression and increased risk of morbidity and mortality (22, 23). Mothers’ lactation can be affected by malnutrition, with risks that exclusive breastfeeding may not be adequate for the first 6 months of life (14). Short adult stature, which is usually due to early childhood malnutrition, is associated with poor educational performance and is a risk factor for delivery complications, cesarean deliveries, and pregnancy-related morbidity and mortality (24, 25). Anemia is estimated to cause some 20% of maternal deaths (26). Anemia also has profound consequences beyond its role in mortality, including diminished work capacity and perhaps depression (27). Women's workload during pregnancy is itself a risk for her and her child's health and nutrition, worsened when she is already malnourished. Infections in women, especially during pregnancy, are common causes of morbidity and mortality, and will be mitigated by improved nutrition.

The combination of malnutrition in mother and child perpetuates the intergenerational transmission of malnutrition. Small mothers have small babies, who may grow up to be small mothers. On the other hand, interrupting this process can create a virtuous cycle: improving maternal nutrition helps future generations to thrive (11, 24).

## Effective interventions

Through a series of systematic reviews of the literature (published as a supplement in 2012 in *Pediatric and Perinatal Epidemiology*) (28), we assessed the efficacy of 17 maternal nutrition interventions. We also evaluated the evidence on the effectiveness of maternal nutrition interventions (i.e. in large-scale operational programs) through a systematic review of published and ‘gray’ literature (noting that rigorous evaluation is rare) (5) and from the country case studies (5–8). Six priority evidence-based interventions were identified in our review that should be implemented now to improve women's nutrition and birth outcomes. These are described in Table 1, together with a summary of the evidence, application, and suggested priority. It can be seen that in most of these the efficacy is established, and there is enough evidence from large-scale programs to support wider use. In no cases are there quick fixes or magic bullets applicable: mitigating the problems needs sustained attention, through systems reaching people at the most local levels; and the capacity developed to do this.

**Table 1:** Feasible evidence-based interventions for improving women's nutrition and birth outcomes in large-scale programs

	Efficacy, based on meta-analyses	Effectiveness of large-scale programs	Applicability
**What works during and before pregnancy**			
To decrease risk of maternal anemia and intrauterine growth restriction (IUGR)/low birth weight (LBW): a) Iron or IFA or b) multiple micronutrient (MMN) supplementation c) Fortification with iron	Moderate quality evidence for impact on maternal anemia (29). MMNs as good as iron–folate for anemia (30), plus increased birth weight (31). Efficacy not assessed.	*Examples*: Nicaragua (5), Thailand, Vietnam (32). Supplementation considered successful, although worst off especially in rural areas may be missed. *Case studies*^a^ : iron supply and distribution was a major constraint at all levels; acceptability and awareness less so. Fortification of staples: mixed results.	Supplementation is universally applicable – should be early intervention in all populations in low- and middle-income countries (LMICs). Flour fortification can be expanded to more countries.
Balanced protein energy supplementation: to increase birth weight and reduce risks of IUGR/LBW and stillbirth	Moderate/high quality evidence for impact on birth weight, greater in undernourished women (33).	*Examples*: Tamil Nadu Integrated Nutrition Project; Madagascar (5). *Case studies*: used when food insecurity exists, such as periodically in Ethiopia; supplements are mandated in India, although coverage varies.	When substantial resources are available; usually targeted; requires considerable logistics.
Iodine fortification of salt (or supplementation in rare cases): to decrease risk of cretinism and improves cognition	High quality evidence for effects on cognitive development (34).	*Examples*: Thirty-six African countries have over 70% coverage of iodized salt (5). *Case studies*: in Ethiopia major supply problems, iodized oil capsules for mothers used; Nigeria high coverage; India varying implementation.	Universally applicable – should be implemented in all populations.
Conditional cash transfers: to provide cash, and a platform for education, supplement provision	Efficacy in terms of access to and use of services, nutritional status and health outcomes; may be attributed to cash or other components (35).	CCTs are implemented in an increasing number of countries, Brazil and Mexico as examples. Evidence in Mexico programme for impact on birth weight. Evidence from Brazil on reduction of infant deaths due to undernutrition. See also (36).	Provide much greater resources than other programs relevant to nutrition: when initiated should be built on.
**What works before pregnancy**			
Increasing age at first pregnancy	Moderate quality evidence that young maternal age is risk for low birth weight and preterm birth; also for maternal anemia (37).	Interventions include legislation preventing marriage before 18 years, cash incentives, outreach programs to prevent harmful traditional practices. Effectiveness not reviewed. *Case studies*: India has legislation and incentives; Ethiopia also has combatting HTP outreach program	Basic intervention in most LMICs – should have legislation and outreach.
Increasing interpregnancy interval (IPI)	Moderate quality evidence that short IPIs are linked with preterm birth, LBW, and early neonatal mortality (38).	Family planning programs, not reviewed by us.	In family planning programs.

a Case studies: see text.

Three of these interventions apply both before and during pregnancy:

1. Supplementation with iron–folic acid (IFA), or iron with other multiple micronutrients (MMNs): this should be a basic intervention in all populations with anemia, along with control of infectious diseases; effective fortification of staple foods such as wheat and corn flour, and rice, may also contribute to improving micronutrient status in different settings.
2. Iodine fortification of salt, or supplementation in rare cases.
3. Balanced protein energy supplementation: for settings of seasonal or chronic food insecurity when substantial resources and adequate logistics are available, usually targeted; these supplements should be fortified with micronutrients.

IFA supplementation during pregnancy, and sometimes before, is covered in many program guidelines. It is known to be efficacious, but effectiveness is hampered by dysfunctional systems for supply and distribution (particularly at community level), low utilization of antenatal care (ANC), a lack of community demand for IFA requiring promotion and counseling, and barriers to access (5, 13, 29). Improvements are needed in all the processes for iron supplementation, including tablet supply and logistics, at each level of implementation, and importantly in the support and counseling to mothers.

Iodine is of profound importance for fetal and postnatal growth and development. Iodized salt provision – one of the big public nutrition success stories – should be supported as a priority to achieve sustained universal coverage. Presently about 70% of households in LMICs are estimated to consume iodized salt, but this figure averages 53% for Sub-Saharan Africa (11), and is only 20% in Ethiopia and 51% in India (13). Moreover, the poorest and most remote places tend to be the least covered (5). Goiter is estimated to affect 10–15% of the population in LMICs, and inadequate urinary iodine 16–40% (11). Continued efforts are needed to adequately iodize the salt supply and put in place regulatory systems to ensure and monitor its quality and coverage.

Provision of balanced protein energy supplements may be expensive and requires developing new distribution systems, especially if targeted. However, these supplements are a critical option when women simply cannot get enough food of the right quality, to attain good nutritional status, and thus adequate nutrition for the developing child. Distribution programs are implemented with food crises (e.g. due to drought in Ethiopia), or in the long-term through routine services, for example as in Tamil Nadu, India (7). Even in the USA, where some 2 million low-income pregnant, postpartum and breastfeeding women considered to be at ‘nutritional risk’ receive vouchers to purchase foods, benefits on birth outcomes have been demonstrated (39).

Three less direct types of interventions (‘nutrition-sensitive’) that affect the nutrition of women and their children are:

1. Legislation and outreach to reduce the numbers of births to teenagers
2. Family planning programs aiming at increasing interpregnancy intervals (IPIs)
3. Conditional cash transfers.

For the first two, strategies include promotion of girls’ education, enforcement of a legal marriage age of 18 years or older and combatting related harmful traditional practices, and promotion of appropriate family planning behavior. WHO has issued detailed guidelines, including strength of evidence for recommendations (40).

Cash transfer programs are expanding rapidly (41), with the primary aim of reducing poverty. They may be conditional on ANC or nutrition education attendance. Further, they can provide a route for direct distribution of fortified food or micronutrient supplements to pregnant women. Finally, by providing the cash to the mother, they empower women to improve health care or food intake for themselves and their children (5). Although the evidence of impact of cash transfer programs on the nutrition of women and children is mixed, these are likely to become of major importance in the future and should be used as a platform for nutrition-specific interventions.

National health insurance programs, recently reviewed by UNICEF (42), at different stages of development in most LMICs will become an important route to support interventions through the health system.

## Platforms

Means of reaching women with these direct and indirect programs are known, and policy decisions that lead to building and strengthening these are essential for achieving targets. This crucial element is often overlooked; aligning interventions with their intended target groups, how to reach these, with estimates of coverage (e.g. by area) and participation (e.g. of those intended within target areas) should be highlighted in planning and monitoring. These platforms, or routes, include:

1. Through the primary health care system and ANC: these are commonly under-resourced and of limited and inequitable coverage. For instance, the percentage of pregnant women receiving at least one ANC visit, and percentage with a visit in the first trimester, was 28% and 6% in Ethiopia, and 64% and 16% in Nigeria; in India we found a range from 80% in Tamil Nadu receiving ANC in the first trimester to 19% in Bihar (7). There are also important within-country inequalities, with interventions delivered through ANC being least likely to reach mothers who are poor and undernourished (43);
2. Through community-based programs that are typically initiated for children who are often brought by their mothers: such programs are expanding and offer new opportunities – a recent review by WHO identified over 60 large-scale or national programs that have been or are being implemented in LMICs (44);
3. Campaigns, such as child health days, during which services such as immunization, deworming, and other selected interventions are provided. These approaches are most appropriate where health service outreach is very limited, and could be used more to reach women who are typically present and can receive interventions such as micronutrient supplementation; interventions delivered through campaigns tend to be more equitable than those delivered in fixed facilities (43);
4. Emergency assistance, e.g. targeting fortified food supplements to pregnant women;
5. Safety net programs, e.g. targeted cash and/or or food supplements.

These different ways of reaching women are best integrated, and opportunities built on, by strengthening existing systems and incorporating priority maternal nutrition interventions. From the case studies, for example in Ethiopia, it is seen that community-based health and nutrition platforms have expanded rapidly. Health Extension Workers and Community Health Volunteers have had substantial impact on mothers’ access to and use of health services and on child nutritional status (45). The social safety net program (PSNP) supports livelihoods, and in times of drought and food insecurity it provides targeted food assistance to women and children.

In India, a number of routes provide income and food support, and incentives for improved use of health care. For example, nutrition related interventions such as prenatal IFA and/or MMN supplementation are integrated with conditional cash transfer programs; the policy is to promote early entry into ANC and hospital delivery as part of the National Rural Health Mission (NRHM). This provides an ideal opportunity to improve maternal and child health by addressing maternal nutrition. Similarly, recent programs such as the Rajiv Gandhi Scheme for Empowerment of Adolescent Girls (SABLA) that target adolescent girls through the Integrated Child Development Services (ICDS) provide an opportunity to improve preconceptional nutritional status, as well as deliver messages related to delaying age at first birth and spacing.

Many of the above-mentioned platforms hold promise, but rigorous and external evaluations are remarkably few. Scaling up these approaches must be accompanied by proper evaluation, which should usually have prospective designs. Decisions are needed to allocate resources to careful evaluations and to facilitate these as a matter of policy, at the risk of otherwise failing to learn why some programs succeed and others fail. This is needed for management decisions on current programs, and for policy decisions on future ones.

## Policy decisions needed

Setting higher priorities and supporting programs for maternal nutrition – especially within the first 500 days – requires recognition of the need, and decisions on action at several levels. First this applies to international policy-makers and their advisors, including leaders of the ‘first 1,000 days’ movement. Other initiatives may be highly relevant, such as ‘Scaling Up Nutrition’ (SUN), the US Global Health Initiative's Feed the Future, which includes women's anemia among its indicators (46, 47), and other nutrition-specific initiatives. Then at the national level, policy decisions are needed by Ministries and Ministers of Health; President's Office or equivalent, for budget and intersectoral issues; high level planning groups (such as the India Planning Commission); and representatives of major donors and implementing agencies. These should ensure that planning, budget allocations, and intersectoral strategies give priority to meeting maternal nutrition needs.

Progress in meeting Millennium Development Goals (MDGs) and the target for child malnutrition – halving the prevalence of underweight children between 1990 and 2015 – depends considerably on the success in reaching the MDGs for women, such as targets for achieving universal access to reproductive health care and reducing the adolescent birth rate.

## What must be communicated effectively

Clear messages need to be communicated to promote policy decisions and resource commitments for planning, implementing and evaluating of large-scale programs. The following suggestions are all based on current knowledge and evidence:

1. Maternal undernutrition in pregnancy can cause restricted intrauterine growth and development, with potential lifelong effects on the child. These include increased vulnerability to mid-life diseases including diabetes and cardiovascular disease, hence premature death; and impaired neurocognitive development whose effects may be irreversible.
2. Policies need to be decided, at high levels, with resource allocations, to prioritize improving women's nutrition, birth outcomes and early infant nutrition. The necessary interventions have been implemented at scale in a number of countries, and these should be expanded and strengthened. Existing guidelines for basic health care during pregnancy, childbirth, postpartum and newborn care (48) already include these interventions, but often these are not given the same priority as those for maternal or child mortality reduction, and a more integrated approach to the provision of maternal and child health and nutrition care is warranted (49). With such an integrated approach, targets for women's and children's nutrition – notably WHO's (anemia and LBW) and MDGs (child underweight or stunting) – could be met, but not otherwise.
3. Three nutritional interventions are of high priority for better resourcing and more effective implementation at scale:
  - IFA supplementation, with infection control, for anemia;
  - further expanding iodized salt use;
  - balanced protein energy supplementation for IUGR and women's malnutrition. Important non-nutritional interventions to improve maternal and child nutrition and health include delaying age of first birth and increasing IPIs. Conditional cash transfer schemes are rapidly expanding and can improve maternal nutrition through multiple mechanisms, empowering women.
4. Key elements of successful scaling up are established from reviewing programs globally and in specific country case studies: these include enabling factors such as strong government leadership, effective coordination, adequate financing, and implementation capacity. Resources have been committed, through the health, social welfare (e.g. women and child sector in India) and other sectors, with support from donors, and decentralized implementation.
5. Scaling up must be accompanied by rigorous, external evaluations, which should ideally be planned before programs are launched, allowing baseline data to be collected. A fraction of the program budget, ideally 5–10%, should be set aside for evaluation activities.
6. International agencies and national authorities need to recognize that neither children nor women's malnutrition – especially in the first 500 days – will be overcome without these commitments. Systems must be strengthened to reach all women, giving access to effective nutrition interventions.

Now that women's nutrition is rising in priority on the international agenda, to the point where goals for women's anemia and for low birth rate reduction are being set, it is crucial to take the next steps – establishing and communicating how to do this, and fostering the necessary policy decisions and resource commitments. The evidence-based interventions described here provide a basis for effective action.

The focus on the key link between the goals for women's and for children's nutrition should be made explicit and highlighted, so that the prerequisite of good maternal nutrition for child growth is well understood and integrated into programs. The WHO goals for children – 40% reduction in stunting (global target 1), 30% reduction in LBW (target 3) – depend substantially on meeting the maternal nutrition targets (50). This includes increasing exclusive breastfeeding in the first 6 months up to at least 50% (target 5) (50).

Good health and nutrition for the mother while pregnant (9 months) and breastfeeding for 6 months (exclusively is the aim) makes this first 15 months of life – roughly 500 days – the most important for her offspring's growth and development. The results of poor nutrition in pregnancy may not be reversed, and pregnancies cannot wait for help. We need to turn the spotlight on women's needs, for their own and for their children's sake, with focus on this earliest period.

There are no shortcuts or magic bullets. There is no escaping the need to build systems that reach all women, and provide access to known interventions that will benefit women's nutrition: both for its own sake, and as an unavoidable requirement in the first 500 days of life.

## Footnotes

1 http://www.thousanddays.org/
